# Intimate partner violence and antenatal care utilization predictors in Uganda: an analysis applying Andersen’s behavioral model of healthcare utilization

**DOI:** 10.1186/s12889-023-16827-w

**Published:** 2023-11-17

**Authors:** Ronald Anguzu, Rebekah J. Walker, Harriet M. Babikako, Kirsten M.M. Beyer, Julia Dickson-Gomez, Yuhong Zhou, Laura D. Cassidy

**Affiliations:** 1 Division of Epidemiology and Social Sciences, Institute for Health and Equity, Medical College of Wisconsin, Milwaukee, US; 2 Center for Advancing Population Sciences (CAPS), Medical College of Wisconsin, Milwaukee, US; 3 Division of General Internal Medicine, Department of Medicine, Medical College of Wisconsin, Milwaukee, US; 4 Department of Child Health and Development Center, School of Medicine, College of Health Sciences, Makerere University, Kampala, Uganda

**Keywords:** Intimate partner violence, Antenatal care, Andersen’s behavioral model, Healthcare utilization, Uganda

## Abstract

**Background:**

Optimal utilization of antenatal care (ANC) services improves positive pregnancy experiences and birth outcomes. However, paucity of evidence exists on which factors should be targeted to increase ANC utilization among women experiencing intimate partner violence (IPV) in Uganda.

**Objective:**

To determine the independent association between IPV exposure and ANC utilization as well as the predictors of ANC utilization informed by Andersen’s Behavioral Model of Healthcare Utilization.

**Methods:**

We analyzed 2016 Uganda Demographic and Health Survey data that included a sample of 1,768 women with children aged 12 to 18 months and responded to both ANC utilization and IPV items. Our outcome was ANC utilization, a count variable assessed as the number of ANC visits in the last 12 months preceding the survey. The key independent variable was exposure to any IPV form defined as self-report of having experienced physical, sexual and/or emotional IPV. Covariates were grouped into predisposing (age, formal education, religion, problem paying treatment costs), enabling (women’s autonomy, mass media exposure), need (unintended pregnancy, parity, history of pregnancy termination), and healthcare system/environmental factors (rural/urban residence, spatial accessibility to health facility). Poisson regression models tested the independent association between IPV and ANC utilization, and the predictors of ANC utilization after controlling for potential confounders.

**Results:**

Mean number of ANC visits (ANC utilization) was 3.71 visits with standard deviation (SD) of ± 1.5 respectively. Overall, 60.8% of our sample reported experiencing any form of IPV. Any IPV exposure was associated with lower number of ANC visits (3.64, SD ± 1.41) when compared to women without IPV exposure (3.82, SD ± 1.64) at p = 0.013. In the adjusted models, any IPV exposure was negatively associated with ANC utilization when compared to women with no IPV exposure after controlling for enabling factors (Coef. -0.03; 95%CI -0.06,-0.01), and healthcare system/environmental factors (Coef. -0.06; 95%CI -0.11,-0.04). Predictors of ANC utilization were higher education (Coef. 0.27; 95%CI 0.15,0.39) compared with no education, high autonomy (Coef. 0.12; 95%CI 0.02,0.23) compared to low autonomy, and partial media exposure (Coef. 0.06; 95%CI 0.01,0.12) compared to low media exposure.

**Conclusion:**

Addressing enabling and healthcare system/environmental factors may increase ANC utilization among Ugandan women experiencing IPV. Prevention and response interventions for IPV should include strategies to increase girls’ higher education completion rates, improve women’s financial autonomy, and mass media exposure to improve ANC utilization in similar populations in Uganda.

## Background

Intimate partner violence (IPV) or behavior that causes physical, sexual or psychological harm perpetrated by an intimate or ex-partner remains a major and pervasive public health problem [1]. Globally, 35% of women are estimated to have ever experienced some form of violence perpetrated by their intimate partner [2]. Sub-Saharan Africa (SSA) has the highest burden of IPV among low and middle- income countries (LMICs), with 45.5% of women experiencing either physical and/or sexual IPV or non-partner sexual violence [3, 4]. In Uganda, 29.9% of women aged 15–49 years and 10.6% of pregnant women report experiencing physical, sexual or emotional violence perpetuated by their intimate male partner within the last 12 months [5]. Comprehensive and multi-institutional approaches to violence prevention may contribute to achievement of Sustainable Development Goal 3 (SDG3) for sustained improvement in women’s and children’s health [6]. IPV is associated with adverse maternal and newborn outcomes among women in SSA [7] that include: preterm births [8, 9], low birth weight (LBW) [10], spontaneous and induced abortion and neonatal mortality [11]. Social problems resulting from IPV include being stigmatized by communities and family [12]. IPV is also associated with negative psychological consequences on IPV survivors such as mental distress [12], depression [13] as well as increasing the risk for HIV acquisition [14].

The World Health Organization (WHO) recommends a minimum of eight focused antenatal care (ANC) visits with the initial visit in the first trimester [15]. According to the most recent Uganda Demographic Health Survey, 60% of women had four or more ANC visits, and 29% had an ANC in their first trimester [16]. Although maternal morbidity and mortality is generally declining in Uganda, it remains high at 375 maternal deaths per 100,000 live births [5, 17, 18]. Evidence shows that attending ANC adequately increases the likelihood of skilled birth attendance [19, 20], and reduces the risk of maternal morbidity and mortality in addition to reducing the risk of adverse birth outcomes such as LBW, stillbirths, neonatal mortality. Positive maternal and child health outcomes (MCH) may be achieved because ANC is a unique opportunity to promote complication readiness and birth preparedness through provision of a range of quality ANC services aimed at recognizing danger signs of pregnancy [21] along the continuum of care [22–25]. These include routine clinical assessments such as blood pressure measurements, diagnostic evaluations like HIV/STI screening, urine testing, blood group typing, obstetric ultrasound scans for fetal growth monitoring, health education and promotion on breast feeding, and birth planning among others [15]. ANC also includes nutrition advice on iron-folic acid supplementation, deworming, and intermittent preventive treatment for presumptive malaria among others [26]. Studies have shown that low ANC utilization is attributed to inadequate social and financial support, long distances to healthcare facilities [27, 28], unintended pregnancies [29] as well as IPV [24, 25, 30].

Studies consistently show that experiencing IPV lowers ANC clinic attendance in SSA countries [31, 32]. This deterrence from utilizing ANC services limits opportunities to receive the benefits that ANC provides especially being the first point of entry into healthcare systems for pregnant persons for potential detection of IPV among survivors during pregnancy [33]. IPV survivors seek help mostly from family members (57%), followed by their spouse/partner’s family (31%), police (16%); however, most women (71.2%) who experience sexual abuse during pregnancy do not seek any help or tell anyone [5]. Healthcare providers are the least sought after for help following IPV (5%) despite the increased risk of adverse birth outcomes, HIV acquisition, and sero-discordance especially among rural women [34, 35].

Uganda clinical guidelines recommends screening for IPV in primary healthcare settings [33]. This underscores ANC clinics as critical settings to leverage promotion of routine IPV screening by healthcare providers [36, 37] as well as creating opportunities to provide or refer IPV survivors to psychosocial support such as counseling. However, IPV is rarely disclosed or reported by survivors due to fear of retaliatory abuse from partners [38, 39], stigma from society or partners’ family and distrust of healthcare workers [40]. ANC visits provide an opportunity for healthcare workers to build rapport with IPV survivors within a trusting environment and provider-patient relationship fostered to encourage disclosure of spousal abuse [41].

Health worker screening for IPV during ANC supports achievement of the SDG3 target 3.1 to reduce the global maternal mortality [42]. In order to address all forms of gender-based violence (GBV) including IPV, the national policy on elimination of GBV in Uganda [43] prioritizes a multi-sectoral approach using integrated IPV prevention and response efforts. This includes investment in training healthcare providers to gain knowledge and skills to detect IPV, improving health facility infrastructure to allow for more privacy during patient-provider interaction among others. Addressing these policy recommendations may improve detection and disclosure of IPV as well as prompt referrals for existing GBV support services.

Existing literature focuses mainly on individual-level, and socio-cultural determinants of healthcare seeking among women and girls exposed to violence in Uganda [44–47]. A knowledge gap exists on how the contribution of environmental-, population- and individual-level factors are linked to perpetration of violent behavior by intimate partners and ANC service use by IPV survivors. The Andersen’s behavioral model (ABM) of healthcare utilization is appropriate when evaluating the contribution of enabling, predisposing, and needs factors on choice, access, and/or use of ANC services or facilities. The current paper adopted the ABM of healthcare utilization [48, 49] to examine how ANC utilization is affected by multi-level factors. Previous research in Uganda used the same framework to contextualize predictors of maternal and mental health service use, contraceptive use, and HIV acquisition [50–53].

For this study, we adopted ABM to identify potential risk/protective factors for ANC utilization from data collected at the national level through the Demographic and Health Survey (DHS) in order to inform response and prevention strategies for violence against women and girls. Therefore, our study objective was to investigate the relationship between IPV and ANC utilization as well as determine the predictors of ANC utilization in Uganda. We hypothesized that the relationship between IPV and ANC utilization would differ among the different groups of ABM factors.

## Methods

### Study design

We analyzed the 2016 UDHS data for this nationally representative, cross-sectional study among women of reproductive age (15–49 years) in Uganda [54]. This survey was conducted by the Uganda Bureau of Statistics (UBOS) with technical support from USAID-supported MEASURE DHS. The UDHS includes questionnaires for individuals and household members including women, men and biomarker surveys [55]. We analyzed the women’s questionnaire survey data from the 2016 Uganda Demographic Health Survey (UDHS) and the 2014 Uganda Bureau of Statistics (UBOS) health facility data [54, 56].

### Data sources

#### 2016 UDHS women’s dataset

The 2016 UDHS dataset is a two-stage stratified cluster survey and the sampling frame includes all census Enumeration Areas (EA) generated by UBOS. In the first stage, 696 accessible EAs were selected, including 162 in urban and 534 in rural areas. In the second stage, a listing of households in each EA was used to select households to be surveyed. Each EA is a geographical area containing an average of 130 households. We used two data files from the 2016 UDHS namely, (i) individual surveys, and (ii) geographic covariate datasets. The survey dataset contains the respondent’s socio-demographic characteristics, and domestic violence items.

#### 2014 UBOS health facility geocode dataset

We extracted geographical coordinates of public health facilities, their HC levels, and geocodes from the 2014 UBOS visualization dashboard [56] into MS Excel. Health facility coordinates identified their location at region, sub-county, and district levels [56]. We exported health facility geocodes into ArcGIS software to compute spatial accessibility as the near point linear distance in meters between EA centroids/clusters and the nearest health facility providing ANC.

#### Uganda healthcare referral system

The national healthcare system in Uganda is comprised of public and private health sectors [57] providing healthcare services through a decentralized system at national, district and health sub-district levels. In Uganda, ANC services are facility-based and provided at health centers (HC) III, HC IV and hospital levels [58]. At the district level, the healthcare referral system is comprised of village health teams (VHTs), health centers (HC) II, HC III and HC IV [59]. The catchment area for populations served by these HC’s increases from fewer than 500, to 100,000 respectively as described elsewhere [60]. These mainly offer basic prevention, promotion and curative services through outpatient and community outreach. All HCs provide ANC services [57]; however, HC IVs provide additional comprehensive emergency obstetrics and newborn care (CEmONC) services [61].

### Study population

**Fig. 1:**
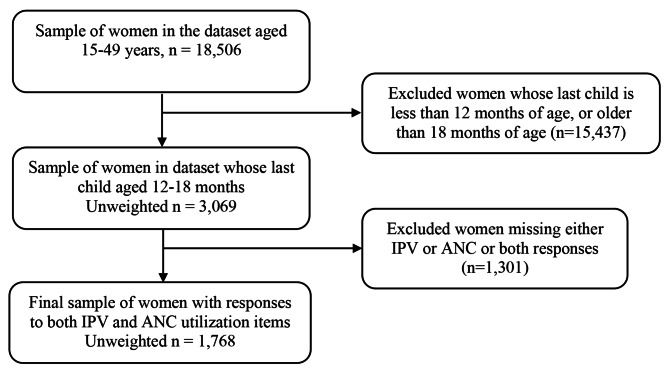
Derivation of our study population

Pregnant women with or without exposure to IPV during pregnancy were identified using the IPV and pregnancy status items selected from the Domestic Violence Module [54] that adopted the Revised Conflict Tactics Scale [62]. The total population of women aged 15 to 49 years in the 2016 UDHS individual recode dataset was 18,506. Figure 1 illustrates the flow chart for the criteria followed to create of our study sample of women aged 15–49 years. Our inclusion criteria were women who responded > = 0 to the item asking about the frequency of ANC service use in the last 12 months. These were women aged 15–49 years whose most recent birth was a live birth, and has a child aged 12–18 months. We excluded: (i) participants who “did not know” (n = 44) or had “missing” responses to number of ANC visits item and (ii) participants who did not respond to all of the items assessing sexual, psychological or physical IPV as shown in Fig. 1. Finally, our study population used for analyses was a sub-population of women in the 2016 UDHS dataset who had responses to both IPV (yes/no) and ANC utilization items (n = 1,768).

### Study measures

#### Outcome variable

The dependent variable was utilization of ANC. This was a count variable measured as the number of ANC visits that study participants had during pregnancy in the last 12 months preceding the UDHS survey. Data on ANC visits was collected among individuals at household level as described elsewhere [16].

#### Key independent variable

Our key independent variable was exposure to at least one form of IPV in the last twelve months (any IPV) preceding this survey interview. This was a composite categorical variable measured as exposure to any: (i) physical, (ii) sexual, and/or (iii) psychological IPV using the revised Conflict Tactics Scale [62]. Similar measures of IPV (any IPV) have been used in previous studies conducted in Uganda [63, 64]. Any IPV form was a categorical variable reported as the proportion of women who reported experiencing at least one of the three IPV forms during pregnancy.

Regarding items measuring physical IPV, respondents were asked whether in the past twelve months, their husband/partner (current or last) ever did the following: *“push you, shook or throw something at you”, “slap you”*, *“fist punch or hit you with something harmful”*, *“kick or drag you”, “strangle or burn you”, “threaten you with knife/gun or other weapon”* and/or *“arm twist or hair pulled”.* A composite binary variable, physical IPV (No/Yes) namely, was constructed No = Never experienced any physical IPV/Yes = Ever experienced at least one form of physical IPV.

Concerning emotional IPV items, participants were asked whether in the past twelve months, their husband/partner (current or last) ever did the following: *“humiliate you”, “threaten you with harm”*, and/or *“insult or made you feel bad”.* These three variables were merged into a binary variable for emotional IPV (No = Never experienced any emotional IPV form/Yes = Ever experienced at least one emotional form of IPV.

In relation to sexual IPV items, participants were asked whether in the past twelve months, their husband/partner (current or last) ever did the following: *physically force you into unwanted sex with him*? *force you into other unwanted sexual acts*? and *physically forced you to perform sexual acts when you did not want to*? A dichotomous composite sexual IPV variable was computed; No = Never experienced any sexual IPV form/Yes = Ever experienced at least one sexual IPV form. Any IPV in the last twelve months was generated as women who reported experiencing either physical, emotional, or sexual violence were coded 1(Yes) while those who reported no to all three types were coded as 0(No).

#### Covariates

**Fig. 2:**
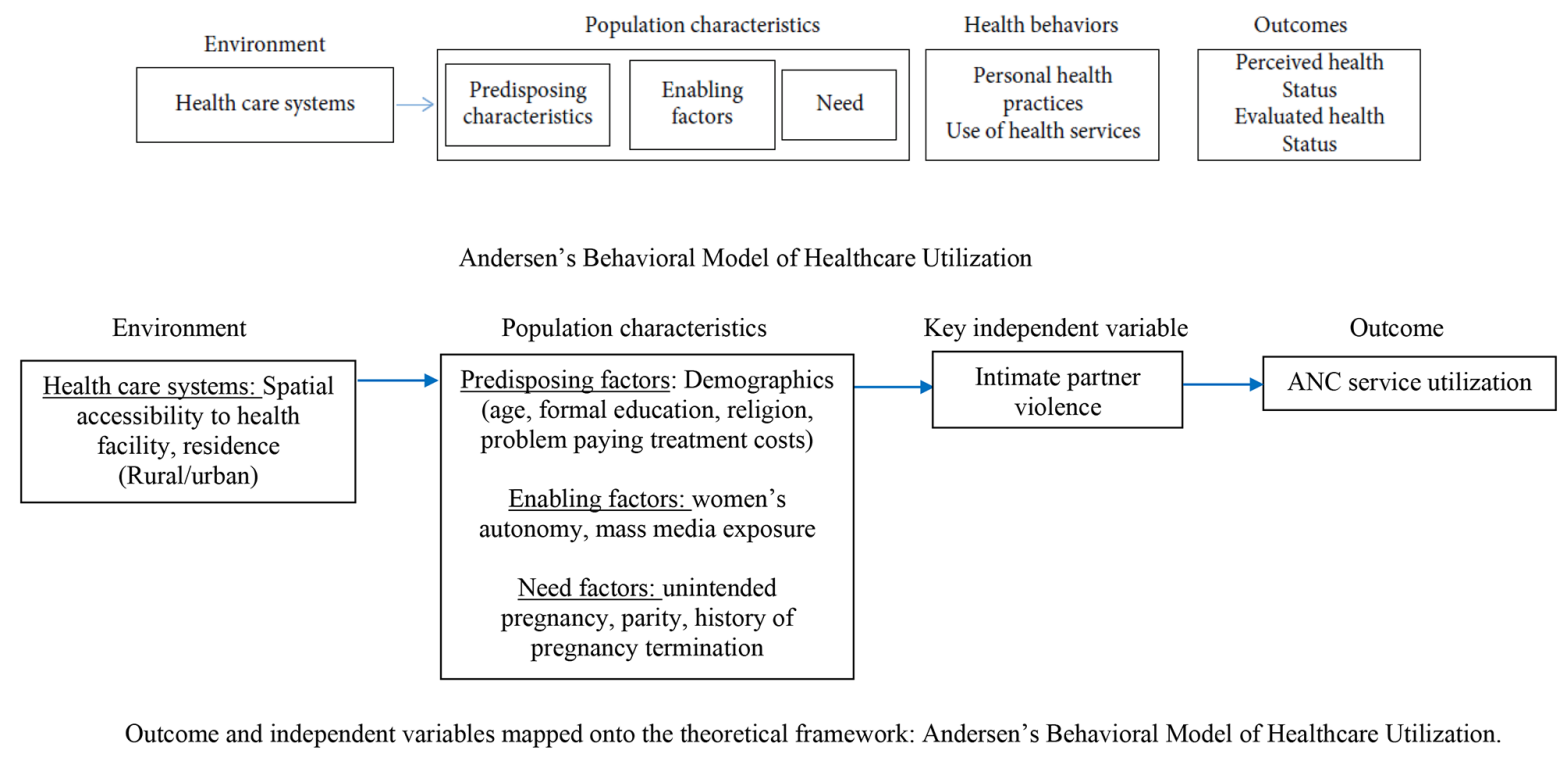
Conceptual framework of the adapted Andersen’s behavioral model of healthcare utilization

Our study adopted ABM domains [65] in order to explore which group/block of factors that explain the association between exposure to IPV and ANC utilization can be targeted by interventions to prevent violence against women. Therefore, ABM informed selection of predictor variables, which were categorized into four blocks, namely, predisposing, enabling, need and healthcare system/environmental factors. Figure 2 demonstrates our conceptual framework of the adapted Andersen’s behavioral model of healthcare utilization. *Predisposing* factors refer to factors that influence an individual’s planned or intended healthcare seeking behavior, specifically ANC utilization in the current study. *Enabling factors* is comprised of factors that may facilitate ANC use or their healthcare seeking behavior. *Need factors* are composed of an individuals’ perceived or actual health or functional needs. The ABM posits that these groups of factors can collectively influence an individuals’ health seeking behavior, particularly ANC utilization. We grouped our covariates based on these ABM domains described in detail as follows.

***Predisposing factors.*** Women’s age was grouped into 15–24, 25–34 and ≥ 35 years of age categories. Education level was categorized into none, primary, secondary and tertiary groups, while religion was categorized into Christian, Muslim, or other groups. We also categorized the item which assessed whether the participant experienced problems getting money for medical treatment (yes/no).

***Enabling factors.*** Women’s autonomy was measured using four items based on prior literature [66, 67], namely whether the respondent: (i) usually decides how to spend respondent’s earnings, (ii) usually make decisions alone about major household purchases, (iii) owns a house alone or jointly, and (iv) owns land alone or jointly. Based on these three items, we generated a composite sum score of women’s autonomy ranging from zero to four, and then categorized the score into three groups: low (none of the items), medium (score 1 to 3), and high (score 4). We generated the mass media exposure variable informed by measurements in prior literature [67, 68]. The three mass media exposure items were: (i) frequency of reading newspapers/magazines, (ii) watching television, and (iii) listening radio. Then, we generated a composite measure ranging from 0 to 3 and categorized into three groups, namely: no exposure (no media exposure), some exposure (one or two media exposures), and full exposure (access to all three mass media items).

***Need factors.*** Items on pregnancy intention assessed whether the respondent wanted their last child (wanted then, wanted later, or wanted no more). We categorized a binary variable as “No” pregnancy wanted later or wanted no more and “Yes” pregnancy wanted then. Parity was a continuous variable defined as the total number of children ever born. ‘Ever terminated pregnancy’ variable assessed whether we the participant has a history of ever terminating a pregnancy (yes/no).

***Healthcare system/Environmental factors.*** Spatial accessibility was categorized as ≥ 5 km (low) or < 5 km (optimal) accessibility based on prior literature [69–72]. Residence was categorized into two nominal categories (rural and urban).

### Data analysis

***Descriptive statistics.*** Data were analyzed using Stata/MPv18.0 and ArcGIS software v16.0 as follows. First, summary statistics were computed for the socio-demographic characteristics and covariates for participants by the mean number of ANC visits. We summarized participant characteristics using frequencies and their respective percentages. Two-sample t-tests, and one-way ANOVA tests were used to assess differences in mean ANC utilization with their corresponding standard deviation (SD) by each participant characteristic with binary, and three or more categories respectively.

**Fig. 3:**
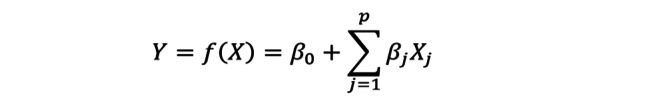
Poisson regression formula

***Unadjusted Poisson regression analysis.*** Second, unadjusted Poisson regression models were run for IPV and each covariate against ANC utilization. Figure 3 illustrates the Poisson regression formula highlighting the outcome variable **Y** as a ANC count (Y/t) with a Poisson distribution with an expected (mean) count of *Yi*, *β*_*0*_ = intercept, and *β*_*j*_ *=* coefficient of independent variable *Xj.* Third, interaction terms run were not statistically significant for the relationship between IPV and ANC utilization by rural/urban residence, therefore, stratified analyses were not conducted. Fourth, there was no collinearity between all covariates. Variables with p-values < 0.2 [73] in unadjusted analyses were included in the adjusted models to determine the; (i) independent association of IPV on ANC utilization, and (ii) predictors of ANC utilization.

***Adjusted Poisson regression analysis.*** Fifth, separate adjusted Poisson regression models were run according to blocks of factors from Andersen’s behavioral model of healthcare utilization as follows: the first model controlled for predisposing factors (Model 1) namely, age, formal education, religion, and experiencing problems paying treatment costs. The second model controlled for enabling factors (Model 2) namely, women’s autonomy, and the degree of exposure to mass media. The third model controlled for need factors (Model 3) namely, unintended pregnancy, parity, and history of pregnancy termination. The fourth model, healthcare system/environmental factors controlled for were rural/urban residence, and spatial accessibility. Lastly, an adjusted Poisson regression model was run for the predictors of ANC utilization (Table 3). We included variables that were statistically significant and variables with p < 0.2 in the unadjusted models into the adjusted model. Statistical significance was determined at *p* < 0.05.

All Poisson regression models were run to generate unadjusted and adjusted coefficients with their 95% confidence intervals by using generalized linear models with link (log) and family (Poisson) commands [74, 75]. Poisson models were run because our outcome variable - ANC utilization in the last 12 months preceding the survey was a count variable [76]. Survey weights were applied to all analyses based on the DHS survey design which accounts for complex survey sampling. Survey commands were run in Stata/MPv18.0 by applying probability/sampling weight (*pweight*), primary sampling unit (*psu*) and stratification used in the sample design (*strata*). *pweight* were computed using the domestic violence weight as described in the DHS methodology. Strata with a single sampling unit were treated as certainty units.

### Ethical considerations

This study was approved by The AIDS Support Organization (TASO) Institutional Review Board (IRB) Ref: TASOREC/083/19-UG-REC-009 and Medical College of Wisconsin (MCW) Ethical Review Committee Ref No: PRO00036397. Permission was obtained from Uganda National Council of Science and Technology (UNCST) Ref: SS 5243. All women who participated in the 2016 UDHS gave informed consent as described elsewhere [54]. Both 2016 UDHS and 2014 UBOS Health facility datasets are publicly available data [56, 77].

## Results

In Table 1, the mean ± SD age of respondents was 35.8 ± 4.8 years while the mean ± SD number of ANC visits (ANC utilization) was 3.7 ± 1.5 visits. Overall, 60.8% of respondents reported experiencing at least one form of sexual, physical and/or emotional IPV (any IPV). In addition, women who experienced any IPV had significantly lower mean ± SD number of ANC visits (3.64 ± 1.41) than those without any IPV exposure (3.82 ± 1.64), p = 0.013. ANC utilization was lowest among women: (i) with problems paying treatment costs (3.58 ± 1.41) when compared to those without problems paying treatment costs (3.82 ± 1.60) at p < 0.001, and (ii) who self-reported their pregnancy as being unintended (3.62 ± 1.50) when compared to those with pregnancies self-reported to be intended (3.75 ± 1.50), p < 0.001 (Table 1). Also, the mean ± SD ANC utilization was significantly lower among women in rural settings compared to urban dwellers [3.63 ± 1.45 vs. 3.99 ± 1.71, p < 0.001] and those with low spatial accessibility to the nearest health facility compared to those with optimal spatial accessibility to the nearest health facility [3.58 ± 1.39 vs. 3.76 ± 1.58, p < 0.001] as shown in Table 1.

**Table 1:** Respondent characteristics by mean antenatal care utilization in Uganda, N = 1,768

Characteristic	N = 1,768 n(%)	Mean (SD) number of ANC visits (ANC utilization)	p-value
**Mean age, years**		35.75 (4.83)	< 0.001
**Parity**		6.67 (2.15)	< 0.001
**Any IPV**			
No	693 (39.2)	3.82 (1.64)	0.013
Yes	1,075 (60.8)	3.64 (1.41)	
**Age group, years**			
≥35	1,004 (56.8)	3.69 (1.60)	0.603
25–34	699 (39.5)	3.74 (1.36)	
15–24	65 (3.7)	4.50 (0.71)	
**Formal education**			
None	408 (23.1)	3.50 (1.47)	< 0.001
Primary	1,128 (63.8)	3.69 (1.47)	
Secondary	186 (10.5)	4.09 (1.71)	
Higher	46 (2.6)	4.70 (1.33)	
**Religion**			
Christian	1,559 (88.2)	3.65 (1.47)	< 0.001
Muslim	196 (11.1)	3.96 (1.68)	
Other	13 (0.7)	3.42 (1.41)	
**Problem paying treatment costs**			
No	777 (43.9)	3.82 (1.60)	< 0.001
Yes	991 (56.1)	3.58 (1.41)	
**Women’s autonomy**			
Low	218 (12.3)	3.63 (1.63)	0.016
Medium	1,217 (68.8)	3.68 (1.46)	
High	333 (18.8)	3.74 (1.53)	
**Mass media exposure**			
Low	513 (29)	3.43 (1.36)	< 0.001
Partial	1,153 (65.2)	3.74 (1.49)	
High	102 (5.8)	4.31 (1.9)	
**Unintended pregnancy**			
No	891 (50.4)	3.75 (1.5)	0.141
Yes	877 (49.6)	3.62 (1.5)	
**History of pregnancy termination**			
No	1,286 (72.7)	3.66 (1.48)	0.072
Yes	482 (27.3)	3.76 (1.54)	
**Residence**			
Urban	277 (15.7)	3.99 (1.71)	< 0.001
Rural	1,491 (84.3)	3.63 (1.45)	
**Spatial accessibility**			
Optimal	995 (56.3)	3.76 (1.58)	< 0.001
Low	773 (43.7)	3.58 (1.39)	

*Column percentage

Table 2 presents the unadjusted and adjusted Poisson regression models for the association between IPV and ANC utilization. In the unadjusted model, IPV exposure was significantly associated with lower ANC utilization (Coef − 0.06; -95%CI -0.11,-0.01, p = 0.028) when compared to no IPV exposure.

**Table 2:** Unadjusted and adjusted Poisson regression models between IPV and ANC utilization, N = 1,768

	Unadjusted		Adjusted models							
			Model 1		Model 2		Model 3		Model 4	
	Coef. (95%CI)	p	Coef. (95%CI)	p	Coef. (95%CI)	p	Coef. (95%CI)	p	Coef. (95%CI)	p
**Any IPV**										
No	Ref		Ref		Ref		Ref		Ref	
Yes	**-0.06 (-0.11, -0.01)**	**0.028**	-0.03 (-0.09, 0.02)	0.227	**-0.02 (-0.04, -0.01)**	**0.021**	-0.05 (-0.11, 0.01)	0.082	**-0.06 (-0.11, -0.04)**	**0.036**
**Age group, years**										
≥35	Ref		Ref							
25–34	-0.01 (-0.06, 0.03)	0.549	-0.01 (-0.06, 0.03)	0.543						
15–24	0.18 (0.02, 0.34)	0.026	0.21 (0.03, 0.39)	0.024						
**Formal education**										
None	Ref		Ref							
Primary	0.04 (-0.03, 0.10)	0.255	0.04 (-0.03, 0.11)	0.223						
Secondary	0.13 (0.04, 0.21)	0.004	0.11 (0.03, 0.20)	0.010						
Higher	0.35 (0.24, 0.47)	< 0.001	0.33 (0.21, 0.44)	< 0.001						
**Religion**										
Christian	Ref		Ref							
Muslim	0.07 (-0.02, 0.15)	0.112	0.05 (-0.03, 0.13)	0.203						
Other	-0.08 (-0.32, 0.16)	0.508	-0.1 (-0.34, 0.13)	0.383						
**Problem paying treatment costs**										
No	Ref		Ref							
Yes	-0.06 (-0.12, -0.01)	0.013	-0.04 (-0.09, 0.01)	0.092						
**Women’s autonomy**										
Low	Ref				Ref					
Medium	0.06 (-0.03, 0.15)	0.169			0.06 (-0.03, 0.15)	0.182				
High	0.13 (0.03, 0.23)	0.012			0.12 (0.02, 0.23)	0.016				
**Mass media exposure**										
Low	Ref				Ref					
Partial	0.08 (0.03, 0.14)	0.002			0.08 (0.03, 0.13)	0.002				
High	0.23 (0.13, 0.33)	< 0.001			0.22 (0.12, 0.32)	< 0.001				
**Unintended pregnancy**										
No	Ref						Ref			
Yes	-0.04 (-0.09, 0.01)	0.141					-0.01 (-0.05, 0.05)	0.986		
**Parity**	-0.03 (-0.04, -0.02)	< 0.001					-0.03 (-0.04, -0.02)	< 0.001		
**History of pregnancy termination**										
No	Ref						Ref			
Yes	0.02 (-0.03, 0.08)	0.417					0.03 (-0.03, 0.09)	0.296		
**Residence**										
Urban	Ref								Ref	
Rural	-0.04 (-0.11, 0.03)	0.232							-0.09 (0.05, 0.54)	0.544
Spatial accessibility, meters*										
<5000 (Optimal)	Ref								Ref	
≥5000 (Low)	-0.06 (-0.12, -0.01)	0.022							-0.11 (-0.03, -0.01)	0.030

*Meters

*Model 1: Predisposing factors*

*Model 2: Enabling factors*

*Model 3: Need factors*

*Model 4: Environment/Healthcare system factors*

### Sequential analyses using ABM domains/blocks

Table 2 shows that exposure to IPV was significantly associated with low ANC utilization when compared to those with no IPV exposure after controlling for enabling factors (Coef. -0.02; 95%CI -0.04,-0.01, p = 0.021) in Model 2, and environmental/healthcare system factors in (Coef − 0.06; 95%CI -0.11,-0.04, p = 0.036) (Model 4). Conversely, the association between IPV exposure and ANC utilization was not statistically significant after controlling for predisposing factors (Model 1) and need factors (Model 3).

### Adjusting using factors in ABM domains

#### Model 1

Among the predisposing factors controlled for, women aged 15–24 years were more likely to.

utilize ANC when compared to women aged ≥ 35 years (Coef 0.21; 95%CI 0.03, 0.39, p = 0.024); women with secondary education (Coef 0.11; 95%CI 0.03, 0.20, p = 0.010), and secondary education (Coef 0.33; 95%CI 0.21, 0.44, p < 0.001) were more likely to utilize ANC when compared to those with no formal education (Table 2).

#### Model 2

After controlling for enabling factors, high autonomy was associated with higher ANC utilization when compared to low autonomy (Coef. 0.12; 95%CI 0.02, 0.23, p = 0.016). In addition, high mass media exposure (Coef. 0.22; 95%CI 0.12, 0.32, p < 0.001) and partial mass media exposure (Coef. 0.08; 95%CI 0.03, 0.13, p = 0.002) were associated with more ANC visits when compared to those with low mass media exposure (Table 2).

#### Model 3

Regarding need factors, each unit increase in parity (total number of children ever born) was.

significantly associated with lower ANC utilization (Coef. -0.03; 95%CI -0.04, -0.02, p < 0.001) (Table 2).

#### Model 4

Among healthcare system/environmental factors controlled for in Model 4, low spatial accessibility to nearest health facilities was associated with lower ANC utilization when compared to optimal spatial accessibility to nearest health facilities (Coef. -0.11; 95%CI -0.03, -0.01, p = 0.030) (Table 2).

Table 3 illustrates the predictors of ANC utilization among women of reproductive age (15–49 years) in Uganda. ANC utilization was more likely among women with: higher education compared to those with no education (Coef. 0.27; 95%CI 0.15,0.39), high autonomy compared to those with low autonomy (Coef. 0.12; 95%CI 0.02,0.23), and partial media exposure compared to those with low media exposure (Coef. 0.06; 95%CI 0.01,0.12).

**Table 3:** Predictors of antenatal care utilization, N = 1,768

Characteristic	Coef. (95% CI)	p-value
Any IPV		
No	Ref	
Yes	-0.03 (-0.08,0.02)	0.244
Age group, years*		
≥35	Ref	
25–34	-0.01 (-0.05,0.04)	0.733
15–24	0.21 (-0.02,0.43)	0.079
**Formal education**		
None	Ref	
Primary	0.03 (-0.04,0.09)	0.419
Secondary	0.08 (-0.01,0.17)	0.058
Higher	**0.27 (0.15, 0.39)**	**< 0.001**
**Religion**		
Christian	Ref	
Muslim	0.06 (-0.02,0.13)	0.160
Other	-0.12 (-0.34,0.10)	0.298
**Problem paying treatment costs**		
No	Ref	
Yes	-0.03 (-0.08,0.02)	0.250
**Women’s autonomy**		
Low	Ref	
Medium	0.06 (-0.03,0.15)	0.193
High	**0.12 (0.02, 0.23)**	**0.019**
**Mass media exposure**		
Low	Ref	
Partial	**0.06 (0.01, 0.12)**	**0.016**
High	0.10 (-0.09,0.19)	0.060
**Spatial accessibility** **		
<5000	Ref	
≥5000	-0.05 (-0.09,0.01)	0.079

*Years, and **meters

## Discussion

To our knowledge, this is one of the first studies to examine the association between IPV and ANC utilization using a nationally representative population of women aged 15 to 49 years in Uganda. Methodologically, we adopted Andersen’s behavioral model of healthcare utilization in order to identify blocks of factors which, when controlled for, affected the relationship between IPV and ANC use. Our study revealed two key findings. First, exposure to any form of IPV was associated with lower ANC visits when compared to women not exposed to IPV after controlling for enabling, and healthcare system/environmental factors respectively. Secondly, the predictors of ANC utilization were attaining higher education, high autonomy levels, and obtaining some (partial) mainstream media exposure. There is a paucity of studies conducted in Uganda that have assessed the relationship between IPV and use of ANC services. However, our findings were in line with previous studies conducted in other SSA countries [30, 78, 79]. For example, women surveyed in Togo who experienced any form of IPV had reduced the number of ANC visits [30], while IPV exposure significantly reduced ANC utilization among women in one province of Mozambique [78]. Similarly, a review representing ten LMICs revealed that experiencing IPV reduced the odds of ANC use [80]. Several potential reasons may explain the association between IPV and ANC utilization.

First, this could be due to low economic autonomy of women experiencing IPV [81] which affects decision making about their finances. This suggests that women who experience IPV may not be able to meet the costs and expenditures needed to obtain ANC. Similarly, as illustrated in our study using the Andersen’s Behavioral Model, high women’s autonomy is an enabling factor that predicted increased ANC utilization. These findings underscore the benefits of promoting economic empowerment like (self)employment [82] guided by theory informed intervention targets. Poverty is another barrier to ANC use especially among women with low-income who may not be able to pay the costs for ANC use. Prior research shows that a woman’s ability to pay for ANC such as their wealth status [83], getting cash payments for their work [84], affects ANC use. This concurred with our study showing that women who had problems paying their treatment costs were less likely to use ANC. Therefore, economic empowerment interventions may increase women’s sources of income and subsequently help women use ANC [85, 86]. Prior research also shows that financial independence may support women in negotiating better treatment within their relationship or leave unsafe circumstances [87]. Conversely, its noteworthy that although empowering women economically is beneficial, it may also increase IPV perpetration because their male partners/spouses may view women’s financial growth as a threat to their control [88].

Secondly, another key factor that potentially explains the negative association between IPV and ANC use could be access to information such as health communication through mainstream media. We revealed that some exposure to mass media increased the likelihood for ANC use. The policy implications of promoting maternal and child health through the media platforms and mobile phone communication are well demonstrated [89–91]. We propose to stakeholders, that they should prioritize the inclusion of women’s preferred or appropriate communication channels during provision of ANC to women experiencing IPV.

The second reason that may potentially explain the relationship between IPV and ANC utilization are the psychological and/or mental health effects of IPV. These include perinatal depression, post-traumatic stress disorder among others which may delay IPV survivors’ decision making to seek [92] and late entry [79] into ANC. The risk of subsequent postpartum depression is likely and may be high among women in Uganda [93–95].

The fourth reason that could explain the association between IPV and low ANC utilization is unintended pregnancies which tends to occur with perpetration of sexual IPV [96–98]. Although our findings showed no association between unintended pregnancy and ANC utilization, prior studies showed that women with sub-optimal ANC visits are more likely to report having an unintended pregnancy [99]. This could be attributed to lack of autonomy in contraceptive decision-making [100] through coercion for pregnancy [101, 102], and contraceptive sabotage by male intimate partners [103, 104] [105]. Our findings call for ANC providers to screen for IPV among pregnant women attending ANC in order to provide or refer IPV survivors for recommended psychosocial and medical management including counseling for family planning according to national policies aimed at the elimination of GBV in Uganda [33, 106].

Finally, another potential reason that may explain the association between IPV and ANC utilization are the healthcare system factors evaluated were rural/urban residence and distance to nearest ANC service providing facility. Prior studies conducted in Uganda [107–111] were in line with our findings showing that shorter distances to the nearest ANC service providing facility were predictors of early or first trimester ANC visits. Related findings were found in other SSA countries which showed that longer distances to the nearest health facilities reduced the number of ANC visits to four or fewer in Ethiopia [112]. Contrary to our findings, prior research conducted in the SSA countries of Ghana [113] and Rwanda [114] found that distance was not associated with optimal ANC utilization. One explanation for this contrary previous finding from the latter countries could be due to implementation of unique national health insurance schemes in Ghana [115, 116], and Rwanda [117, 118] which may address structural barriers to ANC use such as healthcare costs. Regarding the benefits of addressing financial barriers to health service use, prior research from Uganda showed that health insurance increased likelihood for ANC use [119]. The current policy discourse to introduce a national health insurance scheme in Uganda is promising. This may help alleviate barriers to ANC use such as high out-of-pocket payments for ANC use [120].

Future studies should evaluate the impact of IPV on ANC utilization in Uganda using recent data that incorporates current WHO updates to measure eight (8) or more ANC visit cut-offs. Also, future research should assess mediating and moderating effects of predisposing and need factors on the relationship between IPV and ANC utilization. In addition, temporal effects of IPV and ANC utilization should be assessed using longitudinal data in order to draw potential causal inferences. Future research should also use exploratory qualitative methods such as in-depth interviews among women both in communities and health facilities in order to identify underlying factors influencing survivors’ use of ANC as targets for interventions. Policy implications of our findings reveal key targets for policy makers to modify current national policies on elimination of GBV [106] to address/alleviate IPV among pregnant women. Such policy changes should target poverty alleviation, ensure equitable access to ANC, empowerment strategies to improve women’s economic autonomy, ANC education using innovative media and mobile health education platforms to increase ANC utilization.

### Study strengths and limitations

Our study has some strengths. First, we applied survey weights to our analysis making our results generalizable to the population of women with or without: (i) IPV exposure and (ii) ANC attendance. Secondly, we measured exposure to any form of IPV using items that assessed the different IPV forms, namely emotional, sexual, and physical forms of violence in the last 12 months. We consider this composite IPV measure a strength because IPV is known to be under-reported especially in the socio-cultural context of some communities in Uganda where violence against women and girls may be condoned. Our study also had some limitations. First, data analyzed from the 2016 UDHS is drawn from a cross-sectional, population-based survey and therefore causal-temporal inferences should not be deduced. Secondly, recall bias may have affected responses to IPV items in the UDHS survey since these questions probed for potential IPV events that occurred over the last twelve months. Finally, social desirability bias may have occurred and experiencing IPV may be under-reported.

## Conclusions

Mitigation initiatives and future intervention designs aimed at alleviating IPV should consider addressing enabling and healthcare system/environmental factors to increase ANC utilization among Ugandan women experiencing IPV. Comprehensive strategies to alleviate the impact of IPV should aim at improving higher education completion rates for girls, promote women’s financial autonomy via ongoing poverty eradication efforts, use mass media platforms for health promotion, and improving physical accessibility to health facilities in similar populations in Uganda.

## Data Availability

The datasets generated and/or analysed during the current study are available in the DHS Program repository, https://dhsprogram.com/data/available-datasets.cfm for 2016 UDHS data, and the UBOS website accessed via https://ubos.maps.arcgis.com/apps/webappviewer/index.html?id=0fbc1dae45a24f3b89ff61c1f314d375 for the 2014 UBOS health facility geocodes.
